# Biodiversity Monitoring in Impact Assessment Follow-up – Insights From a Large-scale Mine in the Brazilian Amazon Rainforest

**DOI:** 10.1007/s00267-025-02246-7

**Published:** 2025-08-04

**Authors:** Natália Takahashi Margarido, Philippe Hanna, Jos Arts, Larissa Ribeiro Souza, Luis Enrique Sánchez

**Affiliations:** 1 Department of Mining and Petroleum Engineering, Escola Politécnica, University of São Paulo, São Paulo, Brazil; 2 Spatial Planning and Environment, Faculty of Spatial Sciences, University of Groningen, Groningen, The Netherlands; 3 Unit for Environmental Sciences and Management, North West University, Potchefstroom, South Africa

**Keywords:** stakeholder engagement, bauxite mining, ecosystem services, socio-ecological systems, monitoring, follow-up, biodiversity

## Abstract

Biodiversity’s intense decline and the consequences on human wellbeing are of major concern for society. Human activities such as mining, of growing presence in forest areas, threaten biodiversity. Monitoring and reporting for impact assessment follow-up have the purpose to provide and organize information about project impacts, assist decision-making, and improve impact management. However, evidence suggests that biodiversity impacts are not adequately addressed in the impact assessment follow-up process. This paper aims to identify and examine the key elements necessary for securing quality of monitoring and reporting of biodiversity impacts. Based on literature review, key elements of follow-up biodiversity monitoring reports were derived. A longitudinal document analysis was conducted for a bauxite mine operating in a highly biodiverse area, the Amazon, revealing challenges in the application of best practice. Our analysis of the case revealed four emerging issues relevant for improving follow-up practices in the extractive sector: advancing rigor, integration, meaningful stakeholder engagement, and adaptive management. We conclude that improving stakeholder engagement and going beyond compliance requirements, by adopting action plans based on long-term data and a transparent management system, can create a more effective and responsive management of impacts on biodiversity and ecosystem services.

## Introduction

Protecting and restoring nature are matters of growing interest as we face the most intense decline of biodiversity in human history (Brondízio et al. 2019; WWF 2024), threatening life conditions on the planet (Richardson et al. 2023). In 2022, the fifteenth Conference of the Parties (COP) to the United Nations (UN) Convention on Biological Diversity (CBD) adopted the Kunming-Montreal Global Biodiversity Framework (GBF), which established global targets to halt the loss of areas of high biodiversity importance, to restore ecosystems effectively, and to implement conservation measures for critical Biodiversity and Ecosystem Services (B&ES) areas. To meet these goals, not only governments, but also civil society and the private sector, are called upon to implement the proposed actions (CBD/COP/DEC/15/4 2022). Also, the GBF highlights that success will depend on achieving the UN Sustainable Development Goals (SDGs), with an integrative perspective entwining biodiversity and the dependency of human wellbeing on balanced ecosystems, a notion also known as Ecosystem Services (ESs – UN 2015; Costanza et al. 2017).

Mining has been expanding to highly biodiverse regions, with reports indicating that over 40% of large-scale mines were operating in forests (Tibbett 2015; World Bank 2019; Sánchez and Franks 2022). Mining affects ecosystems (Sánchez and Franks Martins et al. 2022) – and consequently ecosystem services (Boldy et al. 2021) and climate dynamics (Odell et al. 2018). Additionally, they often require the construction of associated infrastructure (e.g., access roads), frequently leading to unaddressed cumulative impacts on biodiversity (Siqueira-Gay et al. 2022). Companies’ practices regarding biodiversity have often used targets of No Net Loss (NNL), Net Gain (NG), or Nature Positive. These targets are required by various sector-specific certifications (e.g., ASI, 2022; ICMM, 2024), multilateral financial institutions (IFC 2012; World Bank 2017), and industry groups or investors who consider biodiversity loss to increase risks to their businesses (WEF 2020; TNFD 2023). For a mining project, these targets demand an adequate impact assessment (IA), following the mitigation hierarchy and thus prioritizing to ‘avoid’ and ‘minimize’ impacts to biodiversity values (Cares et al. 2023; Morrison-Saunders and Sánchez 2024).

Impact assessment (IA) is a globally applied tool that assists decision-making by assessing a project’s environmental and social impacts from the entrepreneur’s perspective and determine its feasibility from the standpoint of environmental regulatory agencies. Studies are carried out prior to project implementation (*ex-ante*) and involve collecting data and information to compose a baseline, having impacts identified, predicted, and evaluated, while proposing mitigation measures (Sánchez 2020). After the project has its feasibility attested and licenses are issued, the follow-up stages of implementation and operation begin (*ex-post*). IA follow-up analyses the actual outcomes of assessed impacts by monitoring, evaluation, (adaptive) management, engagement and communication, and safeguarding the governance needed for implementing follow-up (Arts and Morrison-Saunders 2022; Morrison-Saunders et al. 2024).

Monitoring has its roots in the IA *ex-ante* stage, defining a baseline, and during IA follow-up, it produces data and information needed for effective adaptive management (Durning 2012; Moretto et al. 2021; Morrison-Saunders et al. 2024). Monitoring biodiversity has specific challenges to provide effective results, beginning by defining biodiversity (Díaz and Malhi 2022), but also in establishing baseline data in regions with complex ecosystems and highly biodiverse (Gullison et al., 2015; Hoye et al., 2022). Communication during IA follow-up should be able to summarize findings, report impacts management, and transfer knowledge in an accessible manner to all stakeholders (Morrison-Saunders et al. 2023, 2024; Morrison-Saunders and Arts, 2023, 2024). However, in regulatory practice, follow-up reports are usually prepared for the competent agencies, but due to their primary focus on compliance, these reports may not effectively contribute to improved impact management, thus neither deliver results to societal stakeholders. Studies have shown that biodiversity is often not properly addressed in impact assessment – neither *ex-ante* nor *ex-post* – delivering information of limited value to support decision-making (Dias et al. 2019; Mandai et al. 2021; Mäkeläinen and Lehikoinen 2021). Moreover, few longitudinal studies exist that examine monitoring and IA follow-up (Morrison-Saunders et al. 2021).

The aim of this paper is to identify and examine the key elements for securing quality of monitoring and reporting of biodiversity impacts. To this end, we conducted literature review, longitudinal document analysis, and site visits to a bauxite mine in the Brazilian Amazon – a highly diverse region of global importance. Best practices from the literature were categorized, establishing a framework used in the analysis of a practical case, revealing challenges in their application in real-world settings.

## Literature Review

### Biodiversity Monitoring

Biodiversity monitoring is important for environmental management, providing information about the status of biodiversity at multiple spatial scales (from local to planetary), determining intervention needed, and measuring their effectiveness (Lindenmayer et al. 2012). Several technical elements should be considered in the *design* and implementation of monitoring programs to accurately capture information about an ecosystem. First, *statistical rigor* should be adopted in the design to promote detection of trends, optimize field methodologies, and align monitoring frequency with occurrence of the phenomena of interest (Lindenmayer and Likens 2018). Second, the *geographical scale* is important. For instance, adopting the landscape scale, having a less intensive monitoring of many locations, which can also be beneficial to reduce detection errors and avoid misrepresentation (Lamb et al. 2009). This is complementary to the perspective presented by Kühl et al. (2020) of having an *integrative framework* for different scales (temporal or spatial) and types of data, necessary for the creation of a stakeholder network design to long-term and large-scale monitoring. The use of *indicators* or *metrics* to communicate biological diversity is important, and studies comparing indicators of conservation for biodiversity indicate that monitoring of species abundance (Lamb et al., 2009), similarity, and species richness (Santini et al. 2017) performed better. *New technologies* for biodiversity monitoring have become relevant, including the use of remote sensing (Song et al. 2020), drones, and environmental DNA (Allard et al. 2023). In addition, *stakeholder engagement* – such as fostering citizen monitoring (Pocock et al. 2018) by local communities – can be useful in collecting data, while deploying local knowledge and raise environmental awareness.

### Monitoring Biodiversity in Impact Assessment Follow-up

In addition to the biodiversity-specific elements discussed above, IA follow-up professional best practice is laid down in principles established by Morrison-Saunders et al. (2021, 2024), and Arts and Morrison-Saunders (2022), including that: follow-up should be transparent and adaptive, tailored to the regional context, and conducted throughout the entire project lifecycle with clear and predefined performance criteria. Morrison-Saunders et al. (2003) distinguished different regulatory settings – command and control, self-regulation, and public pressure – which may also influence the outcomes of the IA follow-up practices. Command and control refer to a formal compliance-based relationship between regulators and proponents, where authorities are expected to define requirements, monitor results, and oversee implementation. However, regulators do not necessarily have the resources to deliver quality in the follow-up stage. Additionally, the lack of comprehensive regulation regarding IA follow-up may also produce inconsistences on how projects are demanded (Fitzpatrick and Williams 2020). Self-regulation denotes the absence of external mandates, with follow-up depending entirely on the professionals involved. Finally, public pressure involves regulatory structures that are shaped by public concerns, making IA follow-up dependent of affected communities’ mobilization (Morrison-Saunders et al. 2003).

Hardner et al. (2015) argued that biodiversity monitoring in IA should prioritize biodiversity values to be included, and consider direct, indirect, and cumulative impacts. According to Gullison et al. (2015) long-term biodiversity monitoring programs, with the ultimate goal to mitigate project impacts, should: generate meaningful and relevant information with a good cost-benefit ratio, including indicators for processes and results, happening with appropriate frequency, possibly monitoring control sites, and being designed to observe sufficient statistical rigor to support adaptive management.

These principles also resonate with lessons learned from biodiversity monitoring follow-up programs in practice. For example, the study about a pipeline in Peru highlighted the importance of well-defined goals and objectives, collaboration with technical experts and stakeholders, definition of spatial and temporal scales, and systematically prioritizing species or habitats to be studied (Alonso et al. 2013; Dallmeier et al. 2013). The use of proxy variables, where one aspect of biodiversity is used to measure others, is a methodological approach typically used to deal with the technical, time, and economic constraints in project practice. Such approach is exemplified by using forest loss as a proxy to monitor biodiversity changes in forest ecosystems, as applied in evaluating impacts and offsets in a mining project in Madagascar (Devenish et al. 2022). Franke et al. (2022) discussed that the development of a biodiversity conservation tool for monitoring the impacts of a Peruvian copper and zinc mine transformed a 25-year dataset, initially used solely for compliance, into a Biodiversity Action Plan (BAP) for the mine.

A BAP, in accordance with the IFC’s Performance Standards (IFC 2012), should outline a project’s mitigation strategy to achieve NNL, detailing the application of the mitigation hierarchy, and specifying the roles and responsibilities of each stakeholder. Recently, the Aluminum Stewardship Initiative (ASI) required integrating the BAP with existing rehabilitation and mine closure plans, while proactively monitoring the BAP effectiveness and adequacy, evaluating progress towards established goals, and pursuing an early identification of emerging biodiversity risks (ASI 2022).

### Contextualizing Biodiversity Monitoring and Social Interactions

As Ostrom (2009) argued, humans influence environmental resources as part of complex social-ecological systems, and outcomes on sustainability differ because of the interactions among resources units, systems, governance and users. Biodiversity conservation practices have shifted over the past decades from focusing solely on the creation of conservation areas to incorporating local initiatives, ecological restoration, and mechanisms for payment for ESs (Rands et al. 2010). The need for combining conventional science with Indigenous Peoples’ traditional ecological knowledge has been acknowledged in the CBD guidance documents, and reaffirmed in academic literature (Levis et al. 2024).

The ESs-concept also changed, being now framed to emphasize that humans should not only be beneficiaries but also active contributors to the environment, thus adopting a holistic and co-constitutive perspective on nature, potentially leading to more sustainable outcomes (Setten and Brown 2018). This conception aligns with the adoption of the B&ES concept (in opposition as only ESs), as observed in the CBD and in recent corporate certifications (ASI 2022). The assessment of forest restoration (Rosa et al. 2020), and the definition of offset measures, should consider both the areas where offset benefits are generated and where the impacted population resides, in order to produce equitable outcomes (Sonter et al. 2020; Souza et al. 2021).

However, conducting stakeholder engagement, as required, for instance, by World Bank’s (2017) biodiversity monitoring benchmarks, does not guarantee an understanding of the social context that interacts with biodiversity. An integrative framing should also include variables from the social domain to enhance ecosystem management (Schneiders et al. 2012). Geographical and temporal scales may also influence land use differently, and recognizing this, can help to define the most relevant stakeholders to conservation (Allard et al. 2023). Effective monitoring and mitigation thus require a thorough understanding of the social, political, economic, and cultural factors relevant to context. Some authors defend that this process should be community-led and informed by both qualitative and quantitative analyses of the local context (Hanna et al. 2024).

The latter is similar to Slootweg et al. (2001) who proposed to use ‘function evaluation’ – i.e., considering the functioning and interactions between biophysical and social dynamics – as an integrative form of assessing impacts. Such approach also requires a deeper understanding of social values, culture and knowledge that characterizes that society to accurately describe the existing ‘institutional settings’, in order to explain how physical intervention (for instance, mining) would change biophysical settings (in the example, water quality), impacting biophysical functions (a concept similar to ES) and consequently social values (in the example, water usage), and to be able describing impacts of second order or higher to complex cause-effect chains (Slootweg et al., 2001).

## Methodology and Research Context

### Overall Study Approach

We conducted a literature review (presented in “Literature Review”) to establish the key elements of biodiversity monitoring and a longitudinal document analysis over a 17-year period of biodiversity monitoring documents issued by a mining company (see “Longitudinal Analysis of Biodiversity Monitoring at the Juruti Mine”). Additionally, three authors did site visits in August 2022 and June 2024 to familiarize with mining operations and the local and regional context.

Regarding the first step, we conducted a literature review about biodiversity monitoring, in general, and more specifically in the context of IA follow-up. We consulted the Google Scholar database in 2023, combining the terms ‘*biodiversity*’, ‘*monitoring’*, ‘*evaluation’*, ‘*impact assessment’*, ‘*follow-up*’. We used this database to capture not only academic papers, but also professional practice and practical guidance. Documents about single species’ monitoring were not considered, while publications of known relevance for the IA field were also included. Along the process, it was also perceived necessary to gain a deeper understanding of integrated approaches for biophysical and social components in monitoring, so the terms ‘*ecosystem services*’ and ‘*social*’ were also used in combination with the terms used in 2023 for a new round of consultation to the same database in 2024.

The results from the literature review were categorized into key elements of best practice to follow-up programs’, and subsequently into five main topics, which are summarized in Table 1. This allowed for the development of an analytical framework to analyze company reports based on the key elements for biodiversity monitoring and reporting identified in the literature review.

**Table 1:** Key elements for best practice biodiversity monitoring and reporting on IA follow-up, divided by topics

*Topic*	*Key-element*	*Reference*	*Content requirements*
**1. Scope**	**1a. What (to monitor)**	Hardner et al. 2015; Gullison et al. 2015	Scope of the *ex-ante* assessment (i.e., to include indirect and cumulative impacts rather than only direct)
		Schmeller et al. 2017	Key components, functions and processes to monitor
		Dallmeier et al. 2013	Priority species and habitats
		Devenish et al. 2022	Proxy impacts on biodiversity based on vegetation
	**1b. Objectives**	Lindenmayer et al. 2012	Precise objectives for conservation programs.
		Schmeller et al. 2017	Monitoring questions to guide biodiversity monitoring
		Dallmeier et al. 2013	Objectives and goals need to be declared on programs. In the case the goals were: to understand the current situation and the trends of species and habitats of interest.
		Dias et al. 2019	Driving questions to avoid technical quality gaps on fauna monitoring follow-up on large mining projects
**2. Scale**	**2a. Where (to monitor, geographical)**	Dallmeier et al. 2013	Goals and objectives should consider spatial scale
		Lamb et al. 2009	Monitor at landscape scale to reduce detection errors
		Gullison et al. 2015	Include control site when possible
		Barlow et al. 2011	Eco-regional analyses to identify areas with the potential to contain new species; conducting simultaneous research on different taxa to reduce field and laboratory costs.
	**2b. When (to monitor, temporal)**	Dallmeier et al. 2013	Goals and objectives should consider temporal scale
		Lindenmayer and Likens 2018	Should not neglect the repetition of phenomena of interest for monitoring frequency
**3. Approach**	**3a. Standards**	Lindenmayer et al. 2012	Use of appropriate standards to guide activities and provide data for effective long-term monitoring
		Barlow et al. 2011	Development of standards in sampling methods
		Lindenmayer and Likens 2018	Program should include statistical concepts
		del Pozo et al. 2023	Harmonizing monitoring protocols across scales
	**3b. Innovation**	Hoye et al. 2022	Research incentives on the use of molecular biology and remote sensing (image and acoustic methods)
		Song et al. 2020	Remote sensing on landscape, vegetation, soil, water and air conditions
		Allard et al. 2023	Drones, environmental DNA for fauna monitoring
	**3c. (Participation of) Specialists**	Barlow et al. 2011	Investments in local capacity building to make wide monitoring viable
		Dallmeier et al. 2013	Specialists should take part on defining goals and objectives, and on the species and habitats prioritization
		Levis et al. 2024	Conservation efforts should combine conventional science with Indigenous Peoples’ traditional ecological knowledge
	**3d. Stakeholder engagement**	Morrison-Saunders et al. 2023; Morrison-Saunders and Arts, 2023, 2024	Effective public participation, transparency, easy access to reports, continuous access to outputs, inclusion of local knowledge on programs, participatory monitoring
		Gullison et al. 2015	Ongoing stakeholder engagement throughout the monitoring process
		Dallmeier et al. 2013	Stakeholder engagement should take part on defining goals and objectives, and on the species and habitats prioritization
		Torres et al. 2024	Multiple actors are necessary to address the impacts of mining over biodiversity
		Kühl et al. 2020	Integrative structure for monitoring, for both data and programs at the national or regional scale, across taxonomic groups and spatiotemporal scales, shared among stakeholders
		Pocock et al. 2018	Local stakeholder involvement on participatory monitoring, citizenship monitoring
		Hoye et al. 2022	Allow co-creation for biodiversity monitoring design and methodology with the use of citizen science
**4. Contextualization**	**4a. Indicators**	Gullison et al. 2015	Baseline studies integration with long-term monitoring program by adopting consistent methods and indicators across survey sites to accurately assess impacts
			Metrics that generate meaningful and relevant information, with a good cost-benefit ratio; indicators related to processes and results.
		Lamb et al. 2009	Species abundance indices for demonstrating the conservation status during long-term monitoring effectiveness
		Santini et al. 2017	Use of separated metrics for monitoring species abundance, geometric mean of abundance, similarity index and species richness
		Lindenmayer and Likens 2018	Establish trigger points for action to compel institutional and political decision-making
		Stephenson and Carbone 2021	Trigger point indicators for avoiding irreversible biodiversity losses
			Data visualization by maps and charts, contributing to communication, interpretation and decision-making
	**4b. Socio-ecological context**	Allard et al. 2023	Analysis of the social system dynamics (considering social, political, economic and cultural factors) in which biodiversity monitoring is taking place
		Slootweg et al. 2001	Use ‘function evaluation’ as an integrative form for assessing social, biophysical and higher order impacts
**5. Adaptive Learning**	**5a. Adaptative management**	Morrison-Saunders et al. 2021, 2024; Arts and Morrison-Saunders 2022	IA Follow-up should be adaptive
		Dias et al. 2019	Critical analysis and conclusions on fauna monitoring follow-up
		Gullison et al. 2015	Sufficient statistical rigor to support adaptive management
		Dallmeier et al. 2013	Provides for an evaluation of mitigation measures effectiveness
	**5b. Data Management**	Lindenmayer and Likens 2018	Provisions for data sharing (e.g., data curation, quality, format, metadata and intellectual propriety)
		Michener 2015	Data management plan to foster data sharing
		Franke et al. 2022	Use of long-term datasets to contribute for the drafting of overarching BAPs

To enable analysis of the monitoring programs’ processes and contents, we operationalized the key elements of Table 1 into a set of 12 guiding questions (see Table 2, S1). While some elements were aggregated to align with typical themes present in compliance reports – for instance, ‘standard’ and ‘innovation’ were observed as part of the ‘methods’. Other elements were decomposed to address specific ‘indicators’, such as ‘environmental performance’, ‘mitigation’, and ‘occurrence of impacts’. Besides, even though the literature pointed to the dependency of good baselines and impact identification, the questions did not aim to evaluate the quality of the outcomes from previous stages, such as the Environmental and Social Impact Studies (ESIA) in the *ex-ante* assessment. That was a methodological choice given our focus in the follow-up monitoring.

To examine in-depth biodiversity impact management during IA follow-up we used a single ‘exemplar’ case (Flyvbjerg 2006) using key elements and the guiding questions (Table 1, S1). We chose as a case a mine in the Amazon (in Brazil) because: the region is of high biodiversity importance and is under severe pressure (Flores et al. 2024), and mining is an important and expanding economic activity that causes major ecological impacts (Sonter et al. 2023). We selected a bauxite mining, as bauxite extraction affects large land areas and typically takes place in tropical or subtropical forest regions (Murguía et al. 2016). Therefore, we consider the Juruti bauxite mine in the Brazilian Amazon rainforest as relevant internationally to discuss challenges of biodiversity monitoring in IA follow-up.

### Longitudinal Analysis of Biodiversity Monitoring at the Juruti Mine

**Fig. 1:**
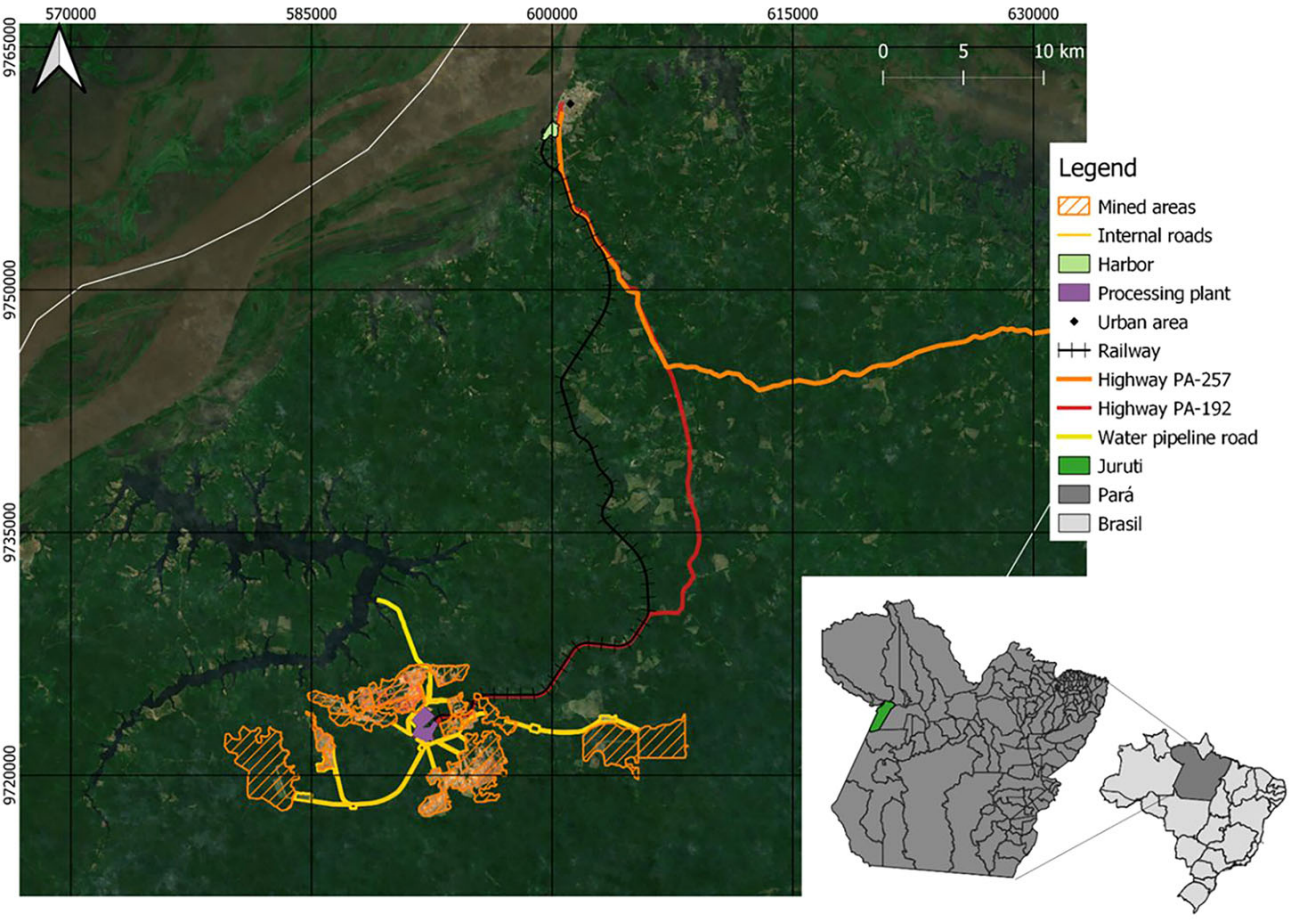
Location of Alcoa Juruti mine

Located in the West of Pará State, the Juruti bauxite mine, owned by Alcoa, began operations in 2009 (Fig. 1). By 2023, its mining capacity was of 7.5 million tons of raw bauxite per year (Alcoa 2023a). The complex includes an open pit mine, processing plant, and storage yards, situated about 55 kilometers from Juruti town. Ore is transported by a company-owned railroad to the Port of Juruti, with public roads being used for transporting goods and employees (Alcoa 2023b).

**Fig. 2:**
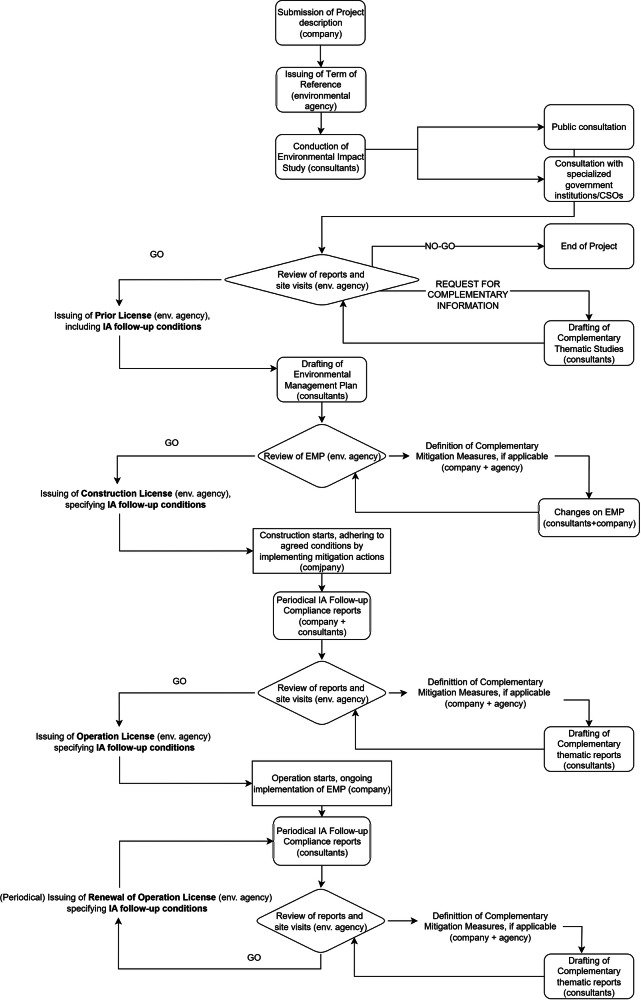
Brazilian Licensing Process (based on National Regulation and Pará State Environmental Agency)

The Pará State Environmental Agency (SEMAS, from the Portuguese - Secretaria de Estado de Meio Ambiente e Sustentabilidade) is responsible for the licensing process, which included the preparation of ESIA in 2002, issuing the construction license in 2006, and the operation license in 2009. Each license comes with conditions established by the regulator, working as a Term of Reference for follow-up stage, which must be implemented and maintained by the company (Hanna et al. 2014). During operations, the mine needs to show annual compliance through monitoring reports, compiled by the company based on the reports produced by their consultants, that are reviewed by SEMAS, who also conduct site visits and may request additional information or complementary mitigation measures, if applicable. Those reports will also be used by SEMAS to decide on the renewal of the operation license in due time. The Brazilian licensing process is presented in Fig. 2. In addition to legal requirements, to comply with ASI standards, the company produced a BAP in 2014, updated in 2023. Impacts on biodiversity were identified and assessed in the BAP for each operational area of the mine, with a total of 22 impacts being managed by 15 follow-up programs (Alcoa 2023b). Each program focuses on mitigating a set of impacts, while some were developed exclusively to manage a given major impact. Terrestrial flora- and fauna-related mitigation is prominent as these biological values are the most impacted by the Juruti mining and exploration activities (CNEC Engenharia S.A. 2004; Alcoa 2023b).

We reviewed follow-up reports issued by Alcoa from 2006 to 2023 to demonstrate compliance with licensing conditions established by SEMAS for both construction and operation stages of the mine. During this period, three consultancies were hired and prepared the reports, relying on their own templates due to the absence of guidelines or standardized reporting formats, given that the responsible environmental agency does not provide them as part of their regulation. This longitudinal approach allowed a historical analysis, and the identification of trends and changes registered in the reports, regarding the five analytical topics defined in Table 1. In 2024, the company changed its approach to data storage and reporting. Four programs (out of the 15 included on the BAP), connected to the most impacted biodiversity, specifically terrestrial flora and fauna, were selected for in-depth analysis.

The *‘Flora Conservation’* program (FL) focuses on vegetation monitoring, which is similar to a baseline study due to its inventory nature in the areas surrounding the mine. This program addresses the impact ‘Modification of floristic composition’ (Alcoa 2023b). In the operation stage, this program was divided in three subprograms: Monitoring of secondary vegetation; Monitoring of threatened species; and Monitoring of *Bertholletia excelsa* (the Brazil nut tree) alongside the road and railway - with the *monitoring of secondary vegetation* subprogram being chosen to focus on here, since it provided for the whole period.

The other selected programs address impacts on terrestrial fauna, namely: ‘Loss of terrestrial fauna’, ‘Decrease of terrestrial fauna populations’, and ‘Decrease of terrestrial fauna diversity’ (Alcoa, 2023b). The *‘Fauna Rescue’* (FR) and *‘Monitoring of Wildlife-Vehicle Collisions’* (WVC) programs, aim to monitor and minimize fauna loss, while the *‘Fauna Monitoring’* program (FM), has an inventorial approach. While the ESIA baseline focused mostly on mammal groups, our analysis of the ‘Fauna Monitoring’ reports focused on amphibians and reptiles, as these were the most frequently recorded groups for fauna loss during operation monitoring.

Table S2 lists the 25 reports we analyzed and indicates the stage that the report is related to (i.e., Construction or Operation) and how they are related to the broader program(s) – for which we use codes: FL for Flora Conservation; FR for Fauna Rescue; FM for Fauna Monitoring; and AR for Annual Report (NB: Wildlife-Vehicle Collisions (WVC) were only reported in the Annual Reports).

## Results and Analysis

The reports were analyzed on how the topics and key best practice elements identified in the literature (Table 1) were addressed in follow-up practice. Based on the guiding questions proposed in Table S1, the degree in which the reports for each program sufficiently met the requirements was critically analyzed and subsequently quantified. These findings are summarized in Table 2 and further discussed in the subsequent sections.

**Table 2:**
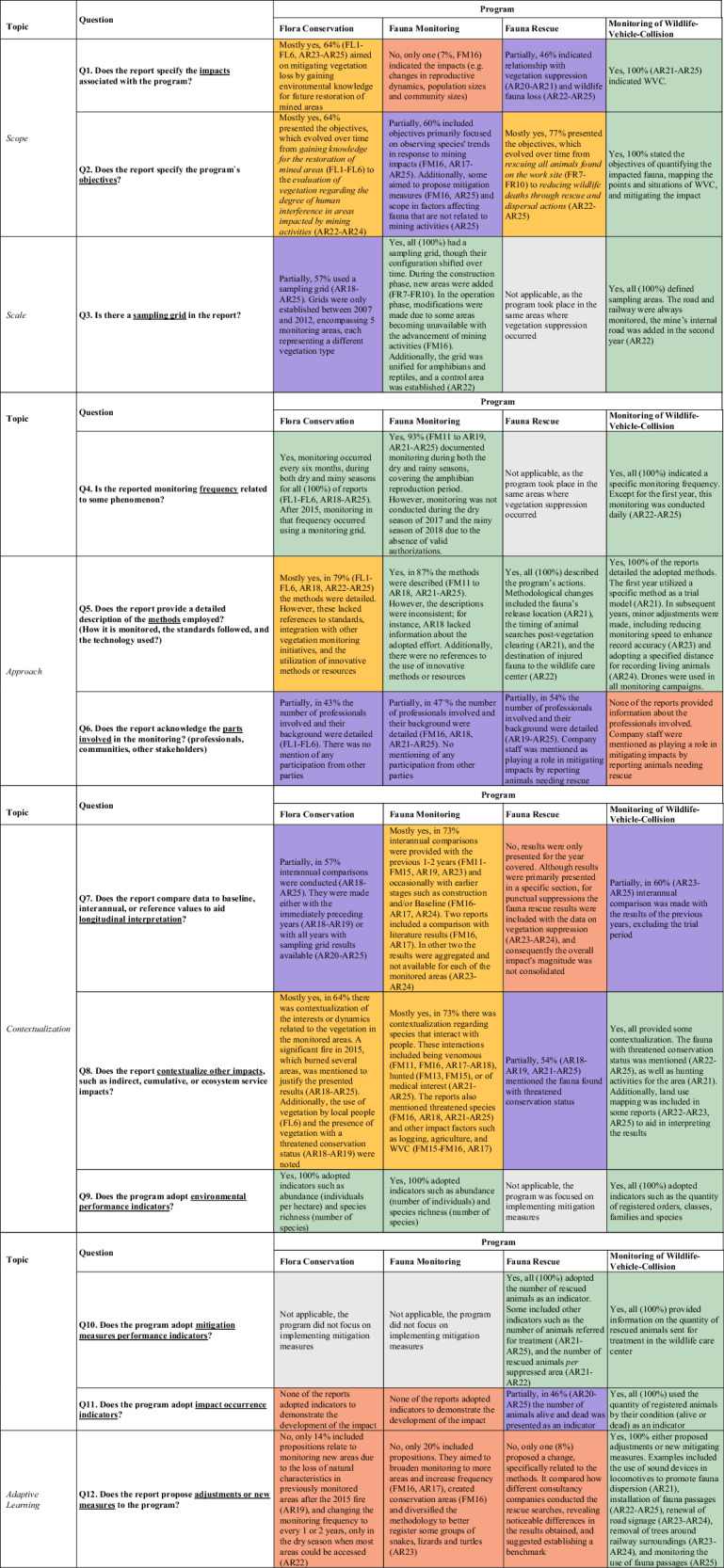
Findings and analysis of the reports related to the four analyzed programs (% of the total number of reports of each program)

NB: The findings and the color coding were divided in: Yes = >80% (); Mostly yes = 61–80% (); Partially = 41–60% (); Mostly not + 21–40% (with no occurrence); No = <20% (); Not applicable (). Reports are numbered consecutively, combined with the codes FL, FR, FM and AR, e.g., FL1 = report # 1, related to the flora conservation program

### Scope

The program of *Flora Conservation* presented a clear change of path to address the impacts. Initially, during early construction stage, the program aimed to know the vegetation composition and investigated the vegetation’s germination characteristics. Monitoring campaigns were held in diverse areas performing qualitative flora inventories (FL1-FL6). Nevertheless, between 2007 and 2012 the program started building one grid to monitor (AR18), and between 2015 and 2023 the ambition became to detect changes by comparison over the years (though not including the 2004 ESIA baseline data), with monitoring being held every six months, as requested by the licensing agency (AR18-AR25). Aligned with this change in approach, reports adopted broader goals, such as evaluating the degree of anthropogenic interference in the monitored areas (AR23-AR24), and thus linking the impact of modification on flora composition with the broader socioecological system, although not in an explicit way.

Only the *Monitoring of Wildlife-Vehicle Collisions* (WVC), the impacts, and program objectives were clearly outlined, as recommended by the literature (Dallmeier et al. 2013; Hardner et al. 2015; Gullison et al. 2015; Schmeller et al. 2017). The *Fauna Monitoring* and *Fauna Rescue* programs showed a trend of improvement, moving from just monitoring occurrence towards actual implementation of mitigation measures. Notably, since 2020 (AR22) *Fauna Rescue* reports established clear objectives of reducing fauna loss, demonstrating a better-defined action-oriented goal.

### Scale

**Fig. 3:**
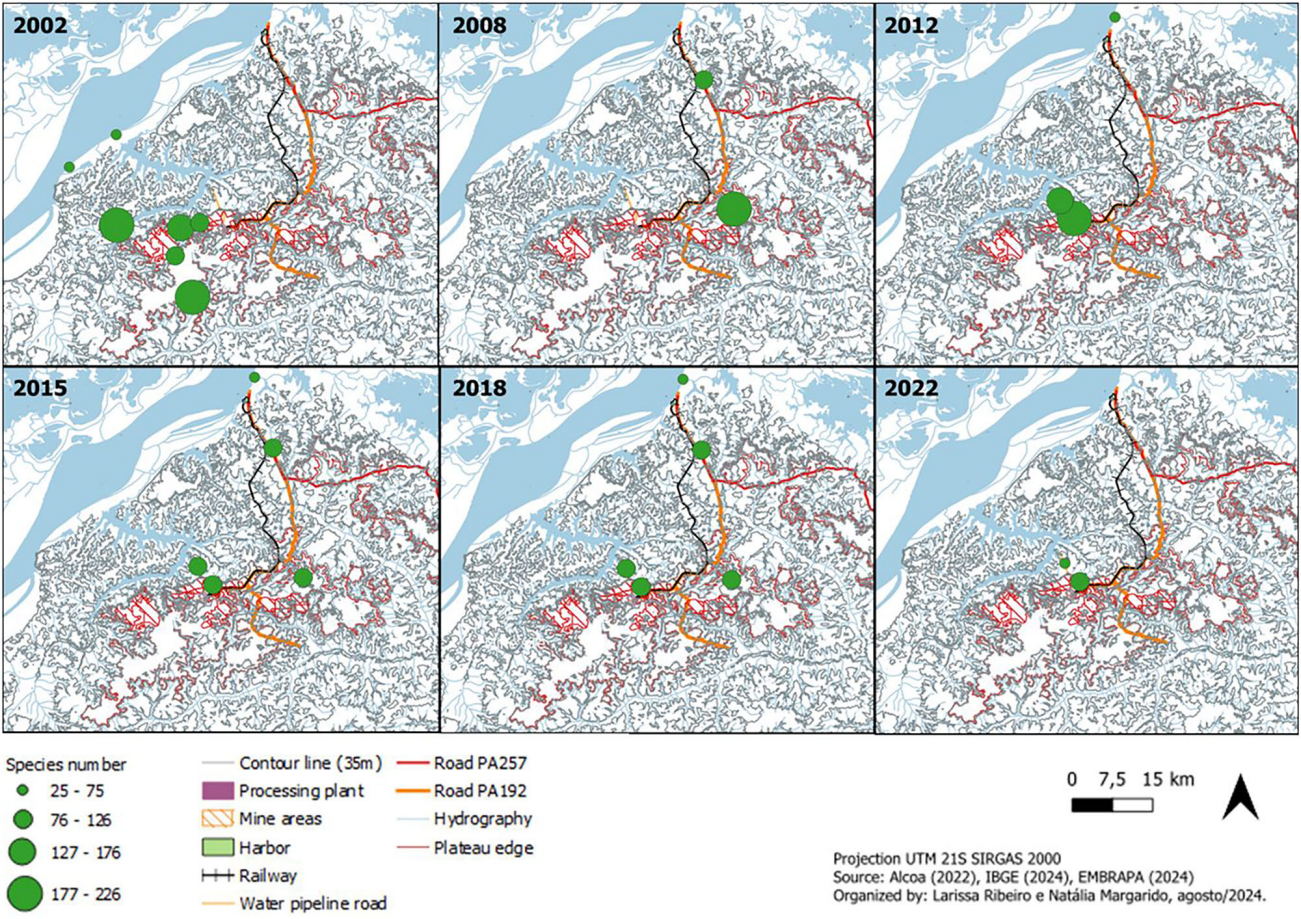
Sampling areas for the Flora Conservation program and results (species richness) over time, illustrating changes of the grid, consequences of fire events on the results, and incomplete data collection due to external factors

The *Flora Conservation* program demonstrated improvements regarding the temporal and spatial scales, with the establishment of a consistent sampling grid and provisions for regular monitoring, in line with the program goals, and with literature (Dallmeier et al. 2013). Practice though indicated challenges that prevented consistent data collection in the newly-defined areas, such as a major wildfire in 2015 (AR19); difficulties in access due to the natural rise of river levels during rainy seasons (AR18, AR20-AR25), and a lack of authorization to access monitoring areas in private properties (AR22, AR24-AR25), evidencing the need of adaptive strategies. This led to a subsequent request to adjust the proposed sampling grid (AR19) and monitoring frequency (AR22), but this was not approved by the licensing agency. Therefore, only limited flora monitoring data were provided in the most recent report (AR25). This inconsistency is displayed in Fig. 3 below, illustrating baseline data of the ESIA and of 5 years for construction and operation stages, using data from the analyzed reports.

The *Fauna Monitoring* program was the only analyzed follow-up monitoring program that employed a control area, as recommended by Gullison et al. (2015). However, during a visit to the mine in 2024, evidence of logging activities was observed in the control area, indicating a potential need to reassess the feasibility of using it as reference for monitoring.

### Approach

The first report of the *Fauna Monitoring* program (FM11) was drafted as supplementary data to fulfill a key deficiency in the ESIA baseline; insufficient data collection about amphibian and reptile groups was collected during the rainy season. Nevertheless, differences in sampling areas, and particularly in monitoring effort, posed challenges in developing subsequent monitoring protocols that integrated well with previous data, an issue also indicated in literature (Barlow et al. 2011; Lindenmayer et al. 2012).

Across the analyzed programs, there was a notable deficiency in the use of statistical resources to describe and support the employed methods, an important feature of monitoring (Lindenmayer & Likens, 2018). Only the *Monitoring of WVC* employed drones. Furthermore, although various authors (Hoye et al. 2022; Morrison-Saunders and Arts 2024) recommend stakeholder engagement and participatory monitoring, such strategies were absent in the reports of the four programs examined, although the company developed several stakeholder engagement initiatives.

### Contextualization

As discussed above, the *Fauna Monitoring* program lacked methodological rigor hampering direct data comparison across years, yet some direct comparisons were presented in the reports, even with misleading interpretations. For instance, abundance and richness were compared by grouping the results according to the different stages of data collection (baseline, follow-up for construction, and follow-up for operation), suggesting that during operation the abundance of animals collected increased (AR 24), while in reality, this relation cannot be scientifically established due to differences in methods, sampling locations, and number of years grouped. Qualitative comparisons, in contrast, were presented more carefully, for instance, describing the major presence of generalist species, relating them to the disturbance in the studied habitat (AR25). Nevertheless, the lack of methodological rigorousness could affect these results too. We did not find evidence that such concerns have been raised by the environmental agency.

Even if many reports did mention species-people interactions (e.g., venomous, hunted, or medical interest), few of them (FM15-FM16, AR17) provided broader regional factors such as logging, agriculture, and WVC. Although one (AR25) stated the aim of understanding the socio-ecological context of impacts on fauna, directly or indirectly related to the mining activity – as recommended by Slootweg et al. (2001) – deeper descriptions of ongoing social dynamics were not used to interpret the results in the reports.

Monitoring WVC employed a comprehensive strategy to monitor animals on the railway, the public highway, and mine-operation roads. The program focused on rescuing at-risk animals or those recently struck by vehicles, while proposing mitigation measures to prevent collisions. Deploying elements of a management system, it utilized indicators to assess environmental quality, effectiveness of mitigation measures, and residual impacts post-mitigation, as recommended by Gullison et al. (2015). Furthermore, the program mapped WVC hotspots (AR21-AR25) while considering land use to enhance proposed mitigation strategies (AR22-AR23, AR25). However, similarly to the other programs analyzed, it did not utilize maps to illustrate impacts, which could improve data accessibility and interpretation (Stephenson and Carbone 2021).

### Adaptive Learning

To mitigate the impacts of vegetation suppression related to different mining activities (e.g., mineral extraction, exploration, and railway maintenance), the *Fauna Rescue* program includes measures to chase away animals prior to these activities, to rescue those with limited mobility, and to search for remaining animals after vegetation suppression. Due to the program’s multifaceted approach (specified per mining activity) multiple consultancy firms were engaged simultaneously. This led to enhancement in conditions to monitor post-vegetation suppression, and to benchmarking methods among consultants regarding spotting animals after bush clearing and thus improving data reported (AR20-AR21). Over the years, the program reported additional initiatives, including the establishment of a wildlife care center at the mine in 2020, reducing the distance for specialized treatment (AR22). In a recent site-visit to the mine, it was noted that the areas surrounding previous release points experienced significant human encroachment, increasing threats to fauna from hunting. Therefore, adjustments were made in the locations where rescued fauna were released back into nature (AR21). All of this exemplifies adaptive management needed to enhance conservation practices (Dallmeier et al. 2013; Morrison-Saunders et al. 2024).

With respect to data management – while the rationale for adjustments in the programs could be well justified, as illustrated by the *Fauna Rescue* program – the reports lacked evidence in demonstrating that proposed changes were supported by data gathered and analysis. The reports presented neither any perspective that the generated data would be shared with other stakeholders, nor integrated with existing or future projects, as recommended in literature (Michener 2015; Lindenmayer and Likens 2018; Franke et al. 2022).

## Discussion

On the basis of the results presented in “Results and Analysis”, considering the best practice information from the literature review on monitoring and follow-up, we identified four issues for discussion: *rigor*, *integration*, *meaningful stakeholder engagement*, which pointed to a fourth issue, insufficiency of *adaptive management* efforts, as clearly visible in the bottom row of Table 2 which scored the poorest results. Each of these issues will be discussed in the next two sub-sections, which are structured around (i) implementation and reporting, and (ii) program design.

### Implementation and reporting

All the issues identified are actually connected and influence each other’s performance. First, the identified *lack of rigor*, particularly evident in some *Fauna Monitoring* reports, stems primarily from insufficient detail in describing fieldwork methods. This omission impeded long-term result comparisons due to methodological incompatibilities (Lindenmayer et al. 2012). This shortcoming, observed in other mining projects (Dias et al. 2019), hampers result comparisons or leads to misinterpretation of impact magnitude, resulting in a lack of critical analysis about the temporal development of the impact. Our findings show that reports were primarily generated for tokenistic compliance, to meet monitoring requirements, thus failing to provide pointers to improve management. Reports were prepared by three different consultancies (with changes over time), which may have contributed to a long-term inconsistency. Additionally, independent verification could improve methodological rigor and facilitate data use for impact management (Wessels et al. 2015; Morrison-Saunders et al. 2024).

Secondly, our findings indicate a lack of reporting *integration* across data, domains, and programs. Reports generally failed to maintain cohesion with previous monitoring or baseline data, contrary to recommendations found in practical guidance, such as Gullison et al. (2015), as well as poor integrated analysis across programs (Franke et al., 2022). As also evidenced in this case, integration between environmental and social domains in IA practice remains challenging globally (Dendena and Corsi 2015). While IA should address indirect and cumulative impacts and adapt monitoring to context (Slootweg et al. 2001; Allard et al. 2023; Morrison-Saunders et al. 2024), our findings suggest that these are gaps in the way monitoring is being interpreted in the reports, but also that integration is hindered by consultancies’ limited scopes, guided exclusively by meeting licensing requirements, that do not necessarily demand for integration. Achieving comprehensive socio-ecological analysis capable of describing impact pathways – as proposed by Slootweg et al. (2001), would necessitate additional resources, not accounted for unless specific guidance for reporting from the environmental agency. Furthermore, improving the capacity of environmental agencies on review reports would also be necessary to address this second issue.

### Program design

Shortcomings in program design were also found in relation to the need for *meaningful stakeholder engagement* in the management of impacts on B&ES. Best practices recommend involving stakeholders throughout all stages (Dallmeier et al. 2013; Morrison-Saunders and Arts, 2023, 2024), seeking to collaborate and even empower participants to obtain meaningful outcomes (Buhmann et al. 2024). For follow-up, it is also considered best practice not only to grant stakeholders access monitoring data and enable discussion of reported results, but also involving stakeholders in data collection, e.g., through citizen science (Pocock et al. 2018), what also facilitates the incorporation of traditional knowledge into monitoring (Morrison-Saunders and Arts 2024), a resource that is underutilized in Brazil (Hanna et al. 2014; Levis et al. 2024). Additionally, literature stresses that stakeholder engagement could promote data sharing (Michener 2015; Lindenmayer and Likens 2018), thus avoiding a plethora of data that does not integrate with other monitoring efforts (McGlone et al. 2020).

Engagement initiatives have been in place for the mine’s impact management, including paid independent technical consultancy to the communities for analyzing reports and monthly meetings to discuss the occurrence of impacts. Those initiatives also include biodiversity topics with more evident social connection, such as the mine rehabilitation program and environmental education activities. However, these efforts were not evident in the four reviewed programs. This finding suggests that, in case the programs are revised, it would probably not be hard to include local communities’ concerns and knowledge. As reported for the Juruti case (Abdala and Veiga 2024), traditional communities who were not originally included in the IA process achieved, after protests, a symmetrical negotiation with the mining company and public authorities, resulting in benefits for all parties involved – enhancing a social-license-to-operate for the company and an integrated assessment of impacts on ecosystem services (encompassing ecological, socioeconomics, cultural and social aspects) to determine financial compensation for communities. Another positive consequence of this process is the improved capacity of the community “to understand and monitor impacts on its land and even to deal with other business interests, such as energy and lumber enterprises” (Abdala & Veiga, 2024, pp.254). Yet, it is also important to consider the unintended side effects of compensation as documented in the literature, which includes in the context of Brazil (Hanna et al. 2016; Sánchez et al. 2018).

This observation substantiates the claim that stakeholder engagement cannot be sought solely for social or biodiversity monitoring purposes separately. The adoption of a B&ES perspective in the monitoring scope (as developed for the financial compensation) along with better, more integrated and rigorous reporting (as recommended above) could be beneficial for this improved understanding. Such understanding could be further leveraged by including community members in the monitoring process during IA follow-up, developing forms of participation tailored to the context, and aiming to reach co-production and co-decision, the highest level of participation (Morrison-Saunders and Arts 2024). Furthermore, B&ES conservation programs could adopt transparent trigger points as operational limits (Brownlie et al. 2013; Lindenmayer and Likens 2018; Stephenson and Carbone 2021). Such approach could lead to operational cessation if thresholds are exceeded, fostering stakeholder trust and enhancing the mine’s social license to operate – thereby related to adaptive management that is stressed in IA follow-up literature (Morrison-Saunders et al. 2021, 2023, 2024).

Finally, *adaptive management*, when reported, lacked explanation of determining factors or links to previous reports. While factors unmentioned in the reports (e.g., possible community grievances or regulatory interactions) might have driven changes, data-derived lessons rarely informed these adjustments. As argued by Morrison-Saunders and Arts (2023, 2024) increased engagement could enhance adaptive management by raising stakeholder awareness about impacts and promote an integrated analysis. That could also promote refinement regarding what is being monitored and where, overcoming eventual weak points in previous IA stages, what usually would occur in the renewal of the operation license in the Brazilian licensing process. Following the concept of adaptive management, the programs should be designed to allow for continuous improvements, a feature that was notably absent in the analyzed programs. They lacked mechanisms to evaluate the impact’s development and to establish thresholds to triggering potential mitigations (e.g., the previously mentioned trigger points). As a result, the programs could be operating for an extended period without effectively improving impact management – as was seen in the Flora Conservation and Fauna Monitoring programs. However, to make any modification in a monitoring program, a formal agreement from the environmental agency is mandatory. Any deviance from the license’s terms and conditions, including monitoring obligations, could be interpreted as regulatory non-compliance.

On a more positive tone, our findings show that the *Monitoring of WVC* obtained higher scores in relation to the proposed questions (Table 2), despite not fully addressing the discussed issues. It is important to mention that this is a relatively recent program, demanded by the environmental agency, and structured from the beginning to be action-oriented. While the environmental agency effectively addressed WVC impacts, its demands for improved follow-up practices in other programs proved to be limited.

In addition to government oversight, the company adheres to international codes of conduct, has its own corporate policy on biodiversity, and is certified by the Aluminum Stewardship Initiative. For that purpose, it is committed to NNL targets for new projects and has to prepare and update Biodiversity Action Plans (BAPs). These actions provide opportunities to foster adaptive management discussion within the company. However, it is important to emphasize that these discussions can also benefit from meaningful, ongoing stakeholder engagement, as well as by bringing them into public forums (Morrison-Saunders and Arts 2024). This example demonstrates that while action-oriented programs can be effective, broader improvements in IA follow-up face significant institutional and procedural barriers, requiring a combination of regulatory pressure and external influence from stakeholders for it to be successful.

## Conclusion

This study analyzed the key elements to secure quality of monitoring and reporting biodiversity impacts for mining operations in highly biodiverse areas. From literature, key best practice elements were derived to guide a longitudinal analysis of impact assessment follow-up reports of a bauxite mine in the Brazilian Amazon. Four programs were analyzed, bringing to light differences and similarities in their trajectories and revealing which elements of effective monitoring and reporting were observed in practice, but also the challenges in applying best practice.

Addressing the four issues identified in our study could improve follow-up practice. We understand these issues to be relevant for the extractive sector in general and for an international audience. Advancing *rigor* (consistent scope, scales, and methods) and *integration* (among programs, data outputs, and environmental and social domains) are important for enhancing reporting in practice. Regarding program design, promoting *meaningful stakeholder engagement* (by incorporating biodiversity and ecosystem services perspective in the monitoring scope) would provide conditions to implement effective *adaptive management* that is needed to preserve biodiversity. Our findings suggest that the most successful reviewed program (Monitoring of Wildlife-Vehicle Collisions) had management system elements and was action-oriented from its inception, thus being able to propose more effective mitigation actions.

We evidence difficulties and opportunities to apply best practice recommendations. The findings suggest two major areas require attention to improve practice. Firstly, improving stakeholder engagement, by going beyond the traditional social umbrella of programs and promoting participation in biodiversity monitoring programs, to improve their outcomes, especially by forwarding focus to impacts on biodiversity that could also affect communities’ livelihoods. Considering biodiversity and ecosystem services in the assessments, as proposed by the Aluminum Stewardship Initiative certification, among other sources, appears to be a valuable approach for implementing a more integrated socio-ecological systems perspective in impact management. Stakeholders could also contribute to determine impact significance and design mitigation measures using local knowledge. However, designing programs that simultaneously align with environmental agency requirements, respect communities’ cultural contexts and interests, and sustain corporate policies, requires carefully orchestrating different interests and organizational cultures. Secondly, it is necessary to move beyond compliance-based management, given that follow-up activities, although legally required, are not always supported by sufficient guidance regarding the procedures (e.g., detailed terms of reference, methodological guidance and oversight) needed to produce consistent results. Improvements could be obtained using the available data collected over the years, and connecting programs by adopting complementary approaches to generate strategic information that can be fed back into a mine’s operation. For such purpose, challenges may arise in the process of recovering and organizing existing data. Nonetheless, both improvements could work synergistically to create more effective and responsive environmental management practices in the extractive sector, as suggested by literature best practice that formed our theoretical basis.

Future research could contribute by expanding longitudinal studies across multiple cases, using the same methods, to enable comparative analysis and discuss the necessary configurations to properly include biodiversity and ecosystem services in impact assessment processes, thereby enhancing decision-making. Our findings indicated the significant role of external pressures and regulations in driving improvements on follow-up programs. Accordingly, further research could investigate the influence of the value chain (mine-to-market) and respective stakeholders on follow-up programs, and examine how such factors leverage the quality of what is being monitored and assessed. Another topic for future research is the regulatory conditions for follow-up, connected to how practice is being developed for each of the key elements identified in the literature. Additionally, future studies could explore spatiality in monitoring, as our findings suggested this is an important issue, as well as how company governance systems can help achieve effective community engagement and how impacts on B&ES are better managed. Furthermore, it would be interesting to explore the secondary impacts of compensation and offset measures from an integrated socio-environmental perspective, especially in such biodiverse areas.

## Supplementary information

Supplementary Information

## Data Availability

No datasets were generated or analysed during the current study.
