# Supplementary material for: Biodiversity Monitoring in Impact Assessment Follow-up – Insights From a Large-scale Mine in the Brazilian Amazon Rainforest
Source: Environ Manage. 2025 Aug 4;75(10):2554–70. doi: 10.1007/s00267-025-02246-7 (PMC12457556; doi:10.1007/s00267-025-02246-7)
Supplement: Supplementary file 1 — Supplementary Information [file 267_2025_2246_MOESM1_ESM.docx]

# Supplementary Information

Table S1 Guiding questions for report analysis, with the second column detailing their link to the categories established in Table 1

| **Questions** | **Topics and key-elements** |
| --- | --- |
| Q1. Does the report specify the impacts associated with the program? | 1. Scope (1a. what) |
| Q2. Does the report specify the program`s objectives? | 1. Scope (1b. objective) |
| Q3. Is there a sampling grid in the report? | 2. Scale (2a. where) |
| Q4. Is the reported monitoring frequency related to some phenomenon? | 2. Scale (2b. when) |
| Q5. Does the report provide a detailed description of the methods employed?(How it is monitored, the standards followed and the technology used?) | 3. Approach (3a. standards, 3b. innovation) |
| Q6. Does the report acknowledge the parts involved in the monitoring? (professionals, communities, other stakeholders) | 3. Approach (3c. specialists, 3d. stakeholder engagement) |
| Q7. Does the report compare data to baseline, interannual, or reference values to aid longitudinal interpretation? | 4. Contextualization (4a. indicators) |
| Q8. Does the report contextualize other impacts, such as indirect, cumulative, or ecosystem service impacts? | 4. Contextualization (4b. socio-ecological context) |
| Q9. Does the program adopt environmental performance indicators? | 4. Contextualization (4a. indicators) |
| Q10. Does the program adopt mitigation measures performance indicators? | 4. Contextualization (4a. indicators) |
| Q11. Does the program adopt indicators related to the occurrence of impacts? | 4. Contextualization (4a. indicators) |
| Q12. Does the program report propose adjustments or new measures to the program? | 5. Adaptive Learning (5a. adaptive management, 5b. data management) |

Table S2 Documents analyzed (FL1 to AR25) and respective programs in each document

| **Stage** | **Document** | **Code** | ***Flora Conservation*** | ***Fauna Monitoring*** | ***Fauna Rescue*** | ***Monitoring of WVC*** |
| --- | --- | --- | --- | --- | --- | --- |
| Construction | Flora Conservation Program - First monitoring campaign Dec 2006 | FL1 | X | - | - | - |
|  | Flora Conservation Program - Second monitoring campaign Mar 2007 | FL2 | X | - | - | - |
|  | Flora Conservation Program - Third monitoring campaign and annual report Jun 2007 | FL3 | X | - | - | - |
|  | Flora Conservation Program - Fourth monitoring campaign Oct 2007 | FL4 | X | - | - | - |
|  | Flora Conservation Program - Fifth monitoring campaign Jan 2008 | FL5 | X | - | - | - |
|  | Flora Conservation Program - Consolidated report 2006/2007 - with sixth monitoring campaign results - Mar 2008 | FL6 | X | - | - | - |
|  | Deforestation and Cleaning Program – Monitoring Deforestation Services – Report n.1 Oct 2006 | FR7 | - | - | X | - |
|  | Deforestation and Cleaning Program – Monitoring Deforestation Services – Report n.2 Mar 2007 | FR8 | - | - | X | - |
|  | Deforestation and Cleaning Program – Monitoring Deforestation Services – Report n.3 Jun 2007 | FR9 | - | - | X | - |
|  | Deforestation and Cleaning Program – Monitoring Deforestation Services – Report n.4 Nov 2007 | FR10 | - | - | X | - |
|  | Program for Inventory and Monitoring of Terrestrial Fauna – Complementary campaign for rainy season Jun 2006 | FM11 | - | X | - | - |
|  | Program for Inventory and Monitoring of Terrestrial Fauna – First monitoring campaign Oct 2006 | FM12 | - | X | - | - |
|  | Program for Inventory and Monitoring of Terrestrial Fauna – Annual report with second monitoring campaign Oct 2007 | FM13 | - | X | - | - |
|  | Program for Inventory and Monitoring of Terrestrial Fauna – Third monitoring campaign Mar 2008 | FM14 | - | X | - | - |
|  | Program for Inventory and Monitoring of Terrestrial Fauna – Consolidated report 2007 - with fourth monitoring campaign - Mai 2008 | FM15 | - | X | - | - |
| Operation | Technical-scientific report (year 8, Aug 2014-Sep 2015), volume 2 - Monitoring of vertebrate fauna and areneofauna in the influence area of the Alcoa project, municipality of Juruti | FM16 | - | X | - | - |
|  | Annual report 2014 Nov 2014 | AR17 | - | X | X | - |
|  | Annual report 2016 Nov 2016 | AR18 | X | X | X | - |
|  | Annual report 2017 Nov 2017 | AR 19 | X | X | X | - |
|  | Annual report 2017/2018 Nov 2018 | AR 20 | X | X | X | - |
|  | Annual report 2018/2019 Nov 2019 + Annex: Consolidated technical report on biotic component monitoring Set 2019 | AR 21 | X | X | X | X |
|  | Annual report 2019/2020 Nov 2020 | AR 22 | X | X | X | X |
|  | Annual report 2020/2021 Nov 2021 + Annex: Fauna monitoring complete report Aug 2021 | AR 23 | X | X | X | X |
|  | Annual report 2021/2022 Nov 2022 + Annex: Fauna monitoring complete report | AR 24 | X | X | X | X |
|  | Annual report 2022/2023 Nov 2023 + Annex: Fauna monitoring complete report Dec 2023 | AR25 | X | X | X | X |
| **Total of reports analyzed for each program** | | **25** | ***14*** | ***15*** | ***13*** | ***5*** |
